# NUMTs Can Imitate Biparental Transmission of mtDNA—A Case in *Drosophila melanogaster*

**DOI:** 10.3390/genes13061023

**Published:** 2022-06-06

**Authors:** Maria-Eleni Parakatselaki, Chen-Tseh Zhu, David Rand, Emmanuel D. Ladoukakis

**Affiliations:** 1 Department of Biology, University of Crete, Voutes University Campus, 70013 Iraklio, Greece; grad751@edu.biology.uoc.gr; 2 Department of Ecology, Evolutionary and Organismal Biology, Brown University, Providence, RI 02912, USA; lei.ctzhu@gmail.com (C.-T.Z.); david_rand@brown.edu (D.R.)

**Keywords:** mtDNA, NUMT, heteroplasmy, biparental transmission, *Drosophila melanogaster*

## Abstract

mtDNA sequences can be incorporated into the nuclear genome and produce nuclear mitochondrial fragments (NUMTs), which resemble mtDNA in their sequence but are transmitted biparentally, like the nuclear genome. NUMTs can be mistaken as real mtDNA and may lead to the erroneous impression that mtDNA is biparentally transmitted. Here, we report a case of mtDNA heteroplasmy in a *Drosophila melanogaster* *DGRP* line, in which the one haplotype was biparentally transmitted in an autosomal manner. Given the sequence identity of this haplotype with the mtDNA, the crossing experiments led to uncertainty about whether heteroplasmy was real or an artifact due to a NUMT. More specific experiments revealed that there is a large NUMT insertion in the *X* chromosome of a specific *DGRP* line, imitating biparental inheritance of mtDNA. Our result suggests that studies on mtDNA heteroplasmy and on mtDNA inheritance should first exclude the possibility of NUMT interference in their data.

## 1. Introduction

Two decades ago, it was observed that fragments of mitochondrial DNA can be located in the nuclear DNA. These sequences have been called NUMTs, which is an acronym for nuclear mitochondrial DNA [1]. NUMTs have been mapped in the nuclear genome of several organisms, such as humans [2], *Daphnia* [3], *Parus* [4], fig wasps [5], *Hydra*, [6] and *Drosophila* [7]. In most cases, NUMTs have the characteristics of pseudogenes, i.e., they can be fragments of gene(s), they might have stop codons and/or frameshift mutations, and increased dN/dS ratio compared to functional mtDNA genes [8].

As expected, the discovery of NUMTs has triggered a discussion on whether they could compromise the interpretation of mtDNA data in phylogenetic studies. It is possible that mitochondrial pseudogenes are co-amplified with real mitochondrial genes, leading to false estimates of coalescent times and intraspecific variation due to different evolutionary rates and modes of inheritance between mtDNA and nDNA [1,9]. Recent studies suggest that the insertion rate of NUMTs can differ among taxa, but generally they tend to be more frequent in taxa with larger nuclear genome sizes [10,11].

NUMTs were thought to be short-sized sequences, with a mean length varying from 100 to 300 bp [12,13], suggesting that sequencing of larger mtDNA fragments followed by searching for stop codons and frameshift mutations could be a way to exclude NUMT sequences from phylogenetic studies [14]. The recent discovery of very large NUMTs in organisms such as bats [15], birds [16], mammals [17,18] including humans [19] indicates that size alone is not sufficient to distinguish between a NUMT and a real mtDNA sequence. Moreover, if the translocation of the mtDNA to the nucleus is recent, it is likely that the mtDNA may have not yet been pseudogenised and therefore, the NUMT sequence might be free of stop codons or frameshift mutations.

Apart from phylogeny, NUMTs can create noise in studies of mtDNA inheritance and heteroplasmy. Given that nuclear DNA is biparentally inherited, a NUMT can be transmitted by both parents and give the erroneous impression that an individual has paternal mtDNA along with true, maternal cytoplasmic mtDNA. A recent study reported biparental transmission of mtDNA in three human families [20] and provided data that the observed pattern was not caused by NUMTs [21]. Some researchers suggested that the possibility of NUMTs had not been exhaustively ruled out in the original study because the observed pattern of biparental transmission of mtDNA can be caused if there are huge mtDNA sequences translocated recently in the nuclear genome (mega-NUMTs) [22,23]. This debate triggered an extensive study in humans which confirmed the presence of mega-NUMTs in the human genome [19].

In this study, we present evidence that a large NUMT has been recently translocated to the nuclear DNA in a *Drosophila melanogaster DGRP* line. We show that this can imitate biparental mtDNA transmission and produce a signal for false heteroplasmy. We also show that this type of NUMT is technically difficult to distinguish from real paternal mtDNA transmission, particularly because it is located in the *X* chromosome and thus it partially imitates maternal transmission.

## 2. Materials and Methods

### 2.1. Crosses

All lines used in the crosses have nuclear chromosomes from *D. melanogaster*. Most of them are mitonuclear replacement lines, i.e., they possess mtDNA from *Drosophila simulans*. Specifically, they carry the *siI*, *sm21*, and *mau12* mitotypes [24,25] that correspond to *siI*, *siII*, and *siIII* mitotypes [26]. A detailed list of the lines used is shown in Table 1. For each cross, 4–5 pairs of flies were used in each vial, while for each type of cross we had at least 10 replicates. All crosses were performed in a standard *Drosophila* medium [27] at 25 °C with a 12 h light period.

### 2.2. DNA Extraction and PCR Assays

For routine PCR assays, DNA was extracted from single flies, as previously described [28]. Before the extraction, the sex of the flies was determined under a stereoscope along with other phenotypic traits, where needed. Each individual was tested for the presence of both maternal and paternal mitotype with primers that are specific to each mtDNA sequence. For long PCR assays, we pooled 10 flies from each of the following lines; *siI*;*w^1118^*, *sm21*;*w^1118^*, *siI;820, sm21;820, DGRP-820*, and *D. melanogaster Oregon R*. DNA was extracted and purified with a standard protocol [29].

For isolation of pure mtDNA from unfertilized eggs, we set up two cages, one with *siI;820* unmated females and one with *sm21;820* unmated females. Cages contained an appropriate egg-laying medium to facilitate the collection of the eggs (5 g sucrose, 2.25 g agar, 50 mL red-fruit juice, and 50 mL H_2_O). Each egg was homogenized in 15 uL lysis buffer (10 mM Tris-HCl pH 8.0, 1 mM EDTA, 25 mM NaCl). One uL Proteinase K 20 mg/mL was added to each sample and then samples were incubated for 2 h at 55 °C. The presence of specific mtDNAs and the absence of specific nDNAs were verified with template-specific primers (Table 2).

For each PCR reaction, we added 1×  *Taq* polymerase buffer (EnzyQuest Biotechnology, IMBB, Heraklion, Greece), 0.4 mΜ of each primer, 1.5 mM MgCl_2_, 0.2 mM dNTPs, 0.4 mg/mL BSA, 0.5 U *Taq* DNA polymerase, 1 uL DNA, and Nanopure H_2_O up to 15 uL. The PCR conditions were: 2 min at 95 °C followed by 35 cycles of 15 s at 95 °C, 15 s at the appropriate annealing temperature for each of the primer sets (Table 2), and 30 s at 72 °C. A final extension step of 5 min at 72 °C followed.

For the long PCR assays, the conditions were the following: 1× KAPA LongRange Buffer (KAPA Biosystems), 0.5 mM of each primer, 1.75 mM MgCl_2_, 0.2 mM dNTPs, 0.4 mg/mL BSA, 0.625 U KAPA LongRange DNA Polymerase, 100 ng of purified DNA, and Nanopure H_2_O up to 25 uL. Two long PCR assays took place, one with the primer pair 7339+/mel11353R and another with the primer pair mel2057F/7597− (Table 2). The PCR conditions for the primer pair 7339+/mel11353R were 5 min at 95 °C followed by 35 cycles of 30 s at 95 °C, 15 sec at 56 °C, and 4.5 min at 68 °C. A final extension step of 6 min at 72 °C followed. The PCR conditions for the primer pair mel2057F/7597− were 5 min at 95 °C, 35 cycles of 30 s at 95 °C, 15 s at 54 °C, and 6 min at 68 °C, and a final extension step of 6 min at 72 °C.

In all PCR reactions, positive and negative controls were used at all times, to check for the specificity of the primers’ binding. For instance, in PCRs with *mel*-specific primers, we used DNA from *si* lines as negative controls and *mel* lines as positive controls. On the contrary, in PCRs with *si*-specific primers, we used DNA from *mel* lines as negative controls and *si* lines as positive controls. All PCR products were visualized on 1% agarose gel stained with EtBr under UV light.

## 3. Results

In a previous study, the mtDNA from 12 *DGRP* lines of *D. melanogaster* was replaced by six different mtDNAs, three from *D. melanogaster* (mitotypes *OreR*, *AutW132*, *Zim53*) and three from *D. simulans* (mitotypes *siI*, *siII*, and *siIII*), creating 72 chimeric lines each containing nuclear DNA from a *DGRP* line and one of the six mitotypes [24]. In this study, we focused on three lines containing the *DGRP-820* nuclear background and one of the following *D. simulans* mitotypes; *siI*, *sm21*, *mau12* [24,25], which are, respectively, *siI*, *siII*, and *siIII* mitotypes [26]. In all three chimeric lines (*siI;820*, *sm21;820*, *mau12;820*), we detected the presence of *D. melanogaster* mtDNA in heteroplasmy with the *D. simulans* mtDNA using species-specific PCR. The rest of the chimeric lines which contained 11 *DGRP* nuclear backgrounds and the three *D. simulans* mitotypes were homoplasmic for the maternal *D. simulans* mitotypes.

Sequencing of a fragment from the *CoxI* gene revealed that the recovered *D. melanogaster* mtDNA sequence (~790 bp) was 99.5% identical with the *Canton-S D. melanogaster* line. They only differed in two synonymous substitutions. Given that putative heteroplasmy has been recurrently observed in all chimeric lines with the *DGRP-820* nuclear background, we hypothesized that it was related to this particular *DGRP-820* nuclear background. In order to investigate this hypothesis, we designed specific sets of crosses starting from the original *DGRP-820* line rather than the chimeric lines, for two reasons, first to repeat step by step the chromosomal replacement in order to locate the factors responsible for the putative heteroplasmy and second to avoid biases regarding the processes that produced the chimeric lines in the first place.

In the first set of crosses, we replaced each of the three major chromosome pairs (*X*, 2nd, 3rd chromosome) of a *w^1118^* nuclear background containing the *siII* mitotype (as *sm21* in [32]) with the corresponding chromosome pair from the *DGRP-820*. We, therefore, created three lines, each containing a different chromosome pair from the *DGRP-820* line and the other chromosome pairs from the *w^1118^* line (Table 3, Figures S1 and S2). We found that only the stable line 1 carrying the *X* chromosome of the *DGRP-820* (*X^DGRP-820^*) appeared as heteroplasmic, while the others (stable lines 2 and 3), carrying the autosomal chromosomes of the *DGRP-820,* were homoplasmic for the maternal *siII* mitotype. This observation suggests that the putative heteroplasmy was associated with the *X^DGRP−820^*, but we could not exclude the possibility that the *siII* mitotype was somehow involved in creating heteroplasmy. For this reason, we repeated the crosses generating a similar set of the three stable lines of Table 3 but using a *w^1118^* line containing the *siI* instead of the *siII* mitotype (Figures S1 and S2, [32]). Apparent heteroplasmy was again related to the *X^DGRP-820^*. We concluded that the *X^DGRP-820^* was solely responsible for the observed heteroplasmy and that the mtDNA was not responsible for this pattern.

In order to specify the locus (or the loci) of the *X* chromosome that was causing presumed heteroplasmy, we designed and performed standard mapping crosses using a line with four phenotypic markers (*y v f mal^bz^*) in the *X* chromosome (Figure S3). The female progeny from these crosses carried a recombinant *X* chromosome, containing some regions from the *X^DGRP-820^* and some from the mapping chromosome, and those regions could be identified phenotypically. We observed that all progeny containing the region between the markers *f* and *mal^bz^* from the *X^DGRP-820^* were putatively heteroplasmic for *mel* and *siII* mitotypes. All the other progeny which did not have this particular region were homoplasmic for the *siII* mitotype. According to FlyBase, the distance between the *f* and *mal^bz^* markers is 3.15 Mb. The locus (or loci) responsible for the presumed heteroplasmy should be located within this region.

With another set of crosses, we investigated whether the putative heteroplasmy was created once and was repeatedly transmitted through the maternal lineage or whether it was emerging at every generation through paternal leakage. For this purpose, we first crossed *mel*; *X^DGRP-820^* females with males *siII*; *FM6/Y* (Figure S4A). All progeny were homoplasmic for the *mel* mitotype. We then crossed *siII*;*FM6*/*w^1118^* females with *DGRP-820* males (Figure S4B). We found that all female progeny were putatively heteroplasmic for the *siII* mitotype from their mother and for the *mel* mitotype from their fathers, while all males were homoplasmic carrying only the maternal mtDNA. We concluded that the putative paternal mtDNA can be transmitted to the progeny carrying the paternal *X^DGRP-820^*.

In order to investigate whether the presumed heteroplasmic females could transmit both their mitotypes to the next generation, we crossed the female progeny from the latter cross (*siII*/*mel*;*FM6*/*X^DGRP-820^*) with *siIII*;*FM6*/*Y* flies (Figure S5). With this cross, we could also investigate if paternal mtDNA leakage occurs, because the fathers carried a different *D. simulans* mitotype (*siIII)*. Based on the *FM6* marker, we could distinguish the individuals possessing the *X^DGRP-8^*^20^ and search for an association between the transmission of heteroplasmy and transmission of the *X^DGRP-820^*. The progeny from this cross that carried the *X^DGRP-820^* (50%) were putatively heteroplasmic for both mitotypes of their mothers. The rest of the progeny were homoplasmic for the *siII* mitotype of their mothers. No paternal mtDNA leakage was observed at any progeny.

**Figure 1:**
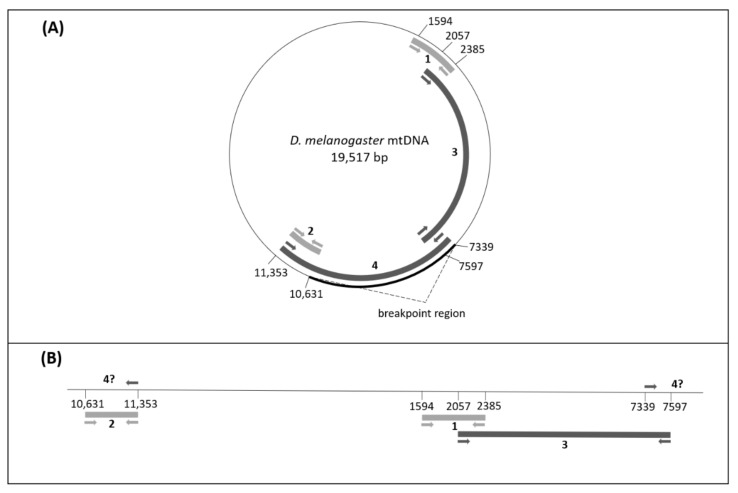
PCR assays designed to detect the *mel* mtDNA and the NUMT. (**A**) The circle represents the *Drosophila melanogaster* mtDNA reference genome. The arrows show the direction of the primers and the thick lines inside the circle represent the potentially amplified fragments, whose coordinates are shown by the numbers. The two short fragments in light grey (*CoxI* 790 bp fragment 1 from 1594 to 2385 and *Cytb* 722 bp fragment 2 from 10,631 to 11,353) represent the PCR fragments that were initially used to detect putative heteroplasmy, while the two long PCR fragments in dark grey lines (fragments 3 and 4) were used to distinguish between true heteroplasmy and NUMT. The bold line on the circle marks the region within which the breakpoint for NUMT insertion resides. (**B**) The NUMT as it should have been inserted in the nuclear genome. The thick lines and the coordinates of the primers and the number of the fragments are similar to part A of the figure. The direction of the primers indicates why the 7339–11,353 fragment was not amplified in the linear molecule. Using the coordinates of the primers and based on the amplified and the non-amplified fragments we estimated the size of the NUMT.

Overall, the results from the crosses showed that a sequence matching the *mel* mitotype is co-transmitted with the *X^DGRP-820^* in the *DGRP-820* chimeric lines. Particularly, the *mel* mitotype showed a biparental mode of transmission, and in cases where both mitotypes were present in males, the *mel* mitotype was only transmitted, while the *si* mitotypes were always transmitted maternally, as expected. There were two possible scenarios for this peculiar pattern. The first scenario suggests that there are actually two mitotypes coexisting in the chimeric lines *siI;820* and *sm21;820*, which are transmitted by both males and females, a scenario that agrees with the biparental transmission of the mtDNA. In this case, there should be a genetic locus on the *X* chromosome that is malfunctioning and therefore is allowing transmission of the paternal mitotype. The second scenario indicates that a NUMT exists on the *X^DGRP-8^*^20^ chromosome that masqueraded as the paternal transmission of the *mel* mitotype, resulting in false heteroplasmy with the *si* mitotypes. Specifically, this would mean that either part or the whole *mel* mtDNA of the *DGRP-820* has migrated to the nucleus, which would explain its transmission pattern. Each scenario could not be easily excluded because they both had contradictory observations. The first scenario is very rarely observed in nature, but at that time there was a recent study reporting biparental inheritance of mtDNA in humans [20]. Moreover, as mentioned above, we saw that the fragment we amplified was almost identical to the *mel* mitotype and contained no stop codons or indel mutations. On the other hand, despite the fact that the existence of a NUMT is more parsimonious than biparental transmission, our sequences were not included in either the annotated NUMTs of the *D. melanogaster* reference genome [7] or in the specific *DGRP-820* genome [33]. The length of annotated NUMTs in *D. melanogaster* varied from 19 bp to 371 bp [7,33], whereas the fragment that we have amplified was more than double the size of the largest NUMT (the PCR fragment we used for the detection of the *mel* mitotype was 791 bp, fragment 1 in Figure 1).

To test these two scenarios, we set up a series of experiments. We first designed another pair of *mel*-specific primers which amplified a 722 bp fragment of the *Cytb* gene (fragment 2), approximately 8 kb away from the amplified fragment 1 (Figure 1). We chose this region for two reasons. First, there were enough nucleotide differences between the *mel* and *si* mitotypes to design specific primers. Second, there was no annotated NUMT in this region. We expected two alternative outcomes from this experiment. If we could not amplify fragment 2 from the *mel* mitotype, it would certainly mean that there was a NUMT in the *DGRP-820* line, because in the case of real heteroplasmy we would be able to amplify any region of the *mel* mtDNA. On the other hand, if we managed to amplify fragment 2, we could exclude neither the biparental mtDNA transmission nor the presence of a large NUMT. We successfully verified the presence of the *mel* mitotype in all putative heteroplasmic progeny tested.

We then asked whether males from the chimeric *DGRP-820* lines (*siI;820*, *sm21;820*) could transmit both their mitotypes (*mel* and *si*) to the next generation. We performed this experiment based on the assumption that if both mitotypes were transmitted then there would be true heteroplasmy and not a NUMT. When crossing these males with *w^1118^* females carrying the *siIII* mitotype (as *mau12* in [32], we observed that we could only detect the *mel* mitotype in the female progeny that also inherited an *X^DGRP-820^* from their father (Figure S6). On the contrary, we did not manage to detect the second paternal mitotype (either *siI* or *siII* depending on the cross) in any of the F1 individuals. This result was an indirect indication that we were actually dealing with a NUMT, because in the case of true heteroplasmy we would expect that the transmission of the two mitotypes could not be discriminated in the sperm.

In another experiment, we attempted to PCR amplify the *mel* mitotype from single unfertilized eggs of females from the chimeric lines *siI;820* and *sm21;820,* in which the *mel* mitotype was found. The rationale of this experiment was based on two facts. Firstly, *D. melanogaster* unmated females lay a small number of unfertilized eggs per day [34]. Secondly, each unfertilized egg carries a large quantity of mtDNA, varying between 8 and 12 million copies, but only a few copies of nDNA, which means that we have pure mtDNA extractions with only little contamination from nuclear DNA [35,36]. As PCR is a semiquantitative method, we expect that the PCR product of mtDNA would be overwhelmingly more than that of the nDNA. To verify this, we used two primer pairs, the pair 7339+/7597−, which exclusively amplified a fragment of both *mel* and *si* mitotypes, and the pair 1002(F)/2653(R), which amplified a fragment on the *X* chromosome (Table 2). We successfully detected mtDNA, but we did not manage to detect nDNA. Hence, we used these preparations to test if the observed *mel* fragments were truly mtDNA or were NUMTs. We tested 20 single unfertilized eggs from *siI;820* or *sm21;820.* We were not able to PCR amplify the *mel* fragment in any of these eggs. This result suggests that the *mel* fragments actually belong to a NUMT, located in the undetectable *X* chromosome of the eggs. The alternative explanation would be that the *mel* fragment was a small minority in the mtDNA pool of the eggs, and therefore undetectable from the *mel*-specific primers we have used. This does not sound plausible, because the *mel* fragment was easily detected in PCRs from whole animal DNA preparations.

**Figure 2:**
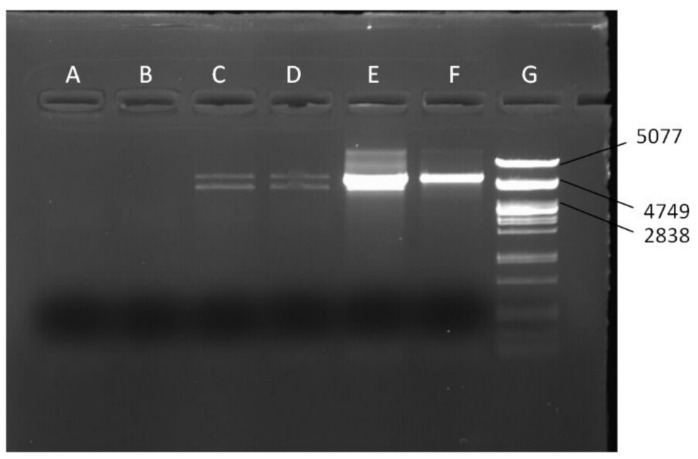
Electrophoresis of the products from long-PCR with primers mel2057F and 7597− (fragment 3). (**A**) *siI;w^1118^*, (**B**) *sm21;w^1118^*, (**C**) *siI;820*, (**D**) *sm21;820*, (**E**) *DGRP-820*, (**F**) *D. melanogaster Oregon R* line, (**G**) λ100 marker, *PstI* digest. (**A**,**B**) are used as negative controls, while (**E**,**F**) are the positive controls. The *mel* product was amplified for lines *siI;820* and *sm21;820*.

**Figure 3:**
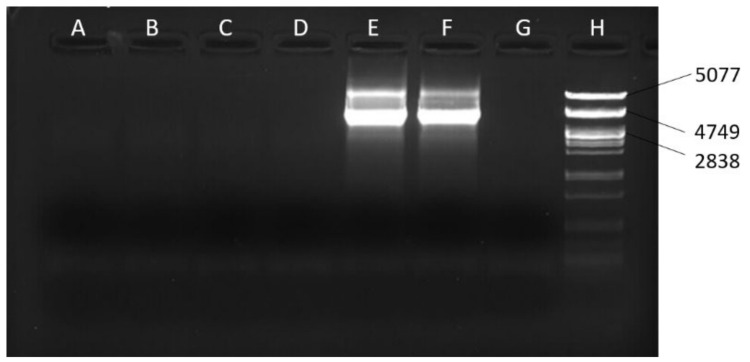
Electrophoresis of the products from long-PCR with primers 7339+ and mel11353R (fragment 4). (**A**) *siI;w^1118^*, (**B**) *sm21;w^1118^*, (**C**) *siI;820*, (**D**) *sm21;820*, (**E**) *DGRP-820*, (**F**) *D. melanogaster Oregon R* line, (**G**) no template control, (**H**) λ100 marker, *PstI* digest. (**A**,**B**) are used as negative controls, while (**E**,**F**) are the positive controls. Absence of the bands in (**C**,**D**) shows that there is no *mel* mtDNA to be amplified in lines *siI;820* and *sm21;820*, a result consistent with the NUMT scenario.

Based on the fact that mtDNA is circular but the NUMTs are linear, we designed long PCR assays to amplify two long overlapping *mel* mtDNA fragments (fragments 3 and 4, Figure 1A,B), with *mel*-specific primers. We tested the chimeric lines *siI;820* and *sm21;820* for the presence of *mel* sequences. We used DNA from the chimeric lines *siI;w^1118^* and *sm21;w^1118^* as negative controls, since they do not contain *mel* mtDNA, and DNA from lines *Oregon R* and *DGRP-820* as positive controls, since they contained *mel* mtDNA, only. If the *mel* sequences were actually NUMTs, we would not recover one of the fragments, because of the incapability of the PCR to amplify the linear fragment (illustrated in Figure 1B). The first 5.5 kb fragment (fragment 3 from 2057 to 7597 bp) was successfully amplified (Figure 2), but the other 4 kb fragment (fragment 4 from 7339 to 11,353 bp) was not (Figure 3). This result suggests that the heteroplasmy observed in the *DGRP-820* line was due to a large NUMT embedded within a 3.15 Mb region of the *X* chromosome, between the *f* and *mal^bz^* genes. The inability to amplify fragment 4 indicates that the junctions of the NUMT are located within this mtDNA fragment. We had already amplified the region between 10,631 and 11,353 bp (fragment 2) using *mel*-specific primers, which is part of fragment 4. Therefore, the breakpoint that linearized the mtDNA should be in a fragment of 3292 bp, between the sites 7339 and 10,631 bp. Given that the length of *D. melanogaster* mtDNA is 19,517 bp and the breakpoint appears to be within 3292 bp, we estimate that the size of the NUMT is at least 16,225 bp.

## 4. Discussion

The attempts to identify NUMTs in the *melanogaster* species group of *Drosophila* revealed nine unique NUMTs in *D. melanogaster* with their size ranging from 19 bp to 371 bp [7,33]. With the exception of *D. simulans*, all other species of this group have more and, on average, larger NUMTs. Interestingly, almost the whole mtDNA has been introgressed in the nDNA in *Drosophila yakuba* [7], which belongs to the *D. melanogaster* species subgroup. The total length of different NUMTs in *D. melanogaster*’s nuclear genome has been estimated at approximately 10.3 kb [9] because NUMTs can be multiplicated after their introgression [13]. The size of the NUMT that we report here is larger than 16.2 kb, which is more than five times the size of the largest NUMT described in the genus, and it is almost the whole mtDNA which is 19,517 kb [37]. This estimate refers to a single-copy sequence. We cannot exclude the possibility that this NUMT has been duplicated in the nDNA, forming a mega-NUMT with several concatemers, as has been proposed by [22]. If present, the duplications must have happened tandemly, because our experiments for NUMT location have shown that it is embedded in a single rather than multiple chromosomal regions. Based on the current observation, the reported insertion rate of mtDNA fragments into nDNA, which has been estimated at 0.12 per million years [7], should be reconsidered. We deduced that the NUMT has been recently introgressed into the nuclear genome, first because its length is much larger than the previously described NUMTs, second because the sequence divergence between the NUMT and the actual mtDNA was negligible, and third because it has only been found in one out of the twelve nuclear *DGRP* backgrounds in the chimeric lines created by [24].

A recent study tried to identify new NUMTs in the sequenced *DGRP* genomes [38] using a bioinformatic approach [33]. This method recovered six NUMTs in all *DGRP* genomes. Two of them had been previously reported [7], while four of them were newly described. It is interesting that this sophisticated method failed to identify three already annotated NUMTs, as well as the one we are describing here. This might have to do with the pipeline they applied, which identifies chimeric reads at the breakpoints of a translocation. These reads may have been discarded as unmapped due to the absence of matching sequences in the reference genome [33]. As difficult as it is to identify a NUMT in the nuclear genome using bioinformatic methods, in our case it was also difficult to distinguish the NUMT from the mtDNA because of their high sequence similarity, perhaps suggesting that the NUMT was recently translocated to the nuclear DNA. This became possible only because the original mtDNA had been replaced by mtDNA from *D. simulans* in the chimeric lines that we initially used. Such polymorphic NUMTs have been systematically identified in other species, as well, such as bats [39], horses [40], pigs [41], and even humans [42,43]. It remains to be tested whether the presence of polymorphic NUMTs suggests that the population of origin (for example, the population that the *DGRP-820* came from) has a higher effective size and therefore can maintain a higher level of polymorphism. Our study brings out an important difficulty in distinguishing NUMTs from true mtDNA, which regards the position of the NUMTs in the genomes. A NUMT located in the *X* chromosome mirrors partially maternal transmission which can be easily confused with the mtDNA transmission. In our case, the combination of the low divergence between NUMT and mtDNA as well as NUMT’s position on the *X* chromosome produced inconclusive results from the crosses and we needed well-targeted experiments to conclude that the putative heteroplasmy we observed was due to a NUMT.

The presence of recent NUMTs in the genomes is not expected to distort the phylogenetic signal of the sequences, because of their sequence similarity with the original mtDNA. It can cause severe problems, though, when studying the inheritance of mtDNA. It is obvious from this study that the heteroplasmy produced by the presence of NUMTs can be easily mistaken for biparental transmission of mtDNA. Such a debate was started by Luo et al. in 2018 in humans [20] and still holds. The evidence they provided for biparental transmission of mtDNA in three human families prompted several researchers to study in depth the validity of this claim. First, Salas et al. [44], reanalyzing the data by Luo et al., estimated that the probability of biparental inheritance is below 10^−37^. Second, Bai et al. [45] showed that, in four human families, heteroplasmic fathers transmitted only one mitotype to their progeny. This observation is similar to what we have observed in the present study, which turned out to be consistent with a NUMT transmission. Third, Wei et al. [46] observed transmission of mega-NUMTs from fathers in seven families, which could erroneously lead to the conclusion that mtDNA is biparentally transmitted. The present study in *D. melanogaster* contributes to the discussion described above for humans. These studies revealed that recent translocations of mtDNA into the nDNA can be more prevalent than previously thought. More importantly, while they cannot disprove the possibility of biparental transmission of mtDNA, they clearly show that such a claim should first exclude the probability that such an observation could be due to NUMTs which are mimicking biparental transmission of mtDNA.

## Figures and Tables

**Table 1:** List of lines used in this study and their mitotypes.

Lines	Mitotype
*mel;DGRP-820*	*mel*
*siI;820*	*siI*
*sm21;820*	*siII*
*siI;w^1118^;+/+;+/+*	*siI*
*sm21;w^1118^;+/+;+/+*	*siII*
*mau12;w^1118^;+/+;+/+*	*siIII*
*D. melanogaster Oregon R*	*mel*
*mel;y v f mal^bz^/ y v f mal^bz^; +/+;+/+*	*mel*
*mel;FM6 B^1^/+;+/+;+/+*	*mel*
*siI;w^1118^;CyO/Sp;TM6B/Dr*	*siI*
*siII;w^1118^;CyO/Sp;TM6B/Dr*	*siII*

**Table 2:** Detailed list of primers used.

Reference	Primers	Sequence (5′ -> 3′)	Specificity	PCR Product Size (bp)	Tm (°C)
[30]	mel1594F	GCTGAATTAGGACATCCTGGAGC	*mel* against *siI, siII, siIII*	791	61
	mel2385R	TCGAGTATCTACATCTATTCCAACG			
Present study	mel10631F	CGAAATTCCCATCCTC	*mel* against *siI, siII, siIII*	722	52
	mel11353R	TTATCAGGGTCTCCCA			
[30]	siI_1737F	TCCTGATATAGCATTTCCA	*siI* against *mel, siIII*	794	58
	siI_2531R	GTTAATCCTCCTACTGTG			
[31]	siII_1737F	CCCTGATATAGCATTCCCG	*siII* against *mel, siIII*	794	58
	siII_2531R	GTTAACCCCCCTACTGTA			
Present study	1588+	GAATTAGGACATCCTGGAGCAT	*siIII* against *mel, siI*, *siII*	786	61
	2374−	GAGTATCAACGTCTATTCCAACTGTG			
Kindly provided by Maria Monastirioti	1002(F)	TCGGAATAAGTTGAAGGATG	*X* chromosome against mtDNA	767	52
	2653(R)	TGCCATCCTGACTGCTCAGC			
Present study	7339+	AAGCATGAGTTAATAAATGAAA	mtDNA universal primers	258	54
	7597−	CCGTTTCTGCTTTAGTTC			
Present study	7339+	AAGCATGAGTTAATAAATGAAA	Used for the long PCR assay	4014	56
	mel11353R	TTATCAGGGTCTCCCA			
Present study	mel2057F	TATTATTATCACTTCCAGTAC	Used with 7597− for the long PCR assay	5540	54
	7597−	CCGTTTCTGCTTTAGTTC			

**Table 3:** Genotypes of three lines produced after the chromosome replacements. Similar lines were produced with the mitotype *siI*.

Line	Genotype
Stable line 1	$siII\left(mel\right);\frac{X^{DGRP-820}}{X^{DGRP-820}};\frac{CyO}{Sp};\frac{TM6B}{Dr}$
Stable line 2	$siII;\frac{w^{1118}}{w^{1118}};\frac{2^{DGRP-820}}{2^{DGRP-820}};\frac{TM6B}{Dr}$
Stable line 3	$siII;\frac{w^{1118}}{w^{1118}};\frac{CyO}{Sp};\frac{3^{DGRP-820}}{3^{DGRP-820}}$

## Supplementary Materials

The following supporting information can be downloaded at: https://www.mdpi.com/article/10.3390/genes13061023/s1, Figure S1: Crossing scheme for the replacement of the *X* chromosome in a *w^1118^* background by the *X* chromosome from the *DGRP-820* nuclear background. The crosses were performed twice, once with a *sm21;w^1118^* line (shown here), and once with a *siI;w^1118^* line (not shown).pdf; Figure S2: Crossing scheme for the replacement of the autosome chromosomes in a *w^1118^* background by the autosome chromosomes from the *DGRP-820* nuclear background. The crosses were performed twice, once with a *sm21;w^1118^* line (shown here), and once with a *siI;w^1118^* line (not shown).pdf; Figure S3: Crossing scheme for mapping the loci responsible for heteroplasmy. The progeny *siIII;FM6/recX^DGRP-820^* were phenotyped to determine which *X^DGRP-820^* regions they contained. The individuals were then PCR-tested for the presence of the *mel* mitotype.pdf; Figure S4: Crosses performed to investigate whether the observed, putative heteroplasmy was created once or whether it was emerging at every generation through paternal leakage. The genotypes of the progeny produced after each cross are shown. (A) All progeny were homoplasmic for the maternal mitotype, while no paternal mtDNA leakage was observed. (B) Progeny that carried the *X^DGRP-820^* carried also both the *mel* and *siII* mitotypes.pdf; Figure S5: Crosses performed to show whether the putatively heteroplasmic females produced by the crosses from Figure S4-B could transmit their presumed heteroplasmy to the next generation. All progeny that inherited an *X^DGRP-820^* from their mothers had also inherited both maternal mitotype sequences (*siII* and *mel*). On the contrary, the rest of the progeny inherited only the *siII* mitotype.pdf; Figure S6: Cross performed to test whether males, putatively heteroplasmic (*mel* and *siII*), with *X^DGRP-820^* chromosome could transmit both their mitotypes to the next generation. Only the *mel* mitotype was transmitted from father, and exclusively to the progeny who also inherited the *X^DGRP-820^*. The same cross was repeated with *siI/mel; X^DGRP-820^/Y* giving the same results (not shown).pdf.
