# Supplementary material for: NUMTs Can Imitate Biparental Transmission of mtDNA—A Case in Drosophila melanogaster
Source: Genes (Basel). 2022 Jun 6;13(6):1023. doi: 10.3390/genes13061023 (PMC9222939; doi:10.3390/genes13061023)
Supplement: Supplementary file 1 [file genes-13-01023-s001.zip › Figure S1.pdf]

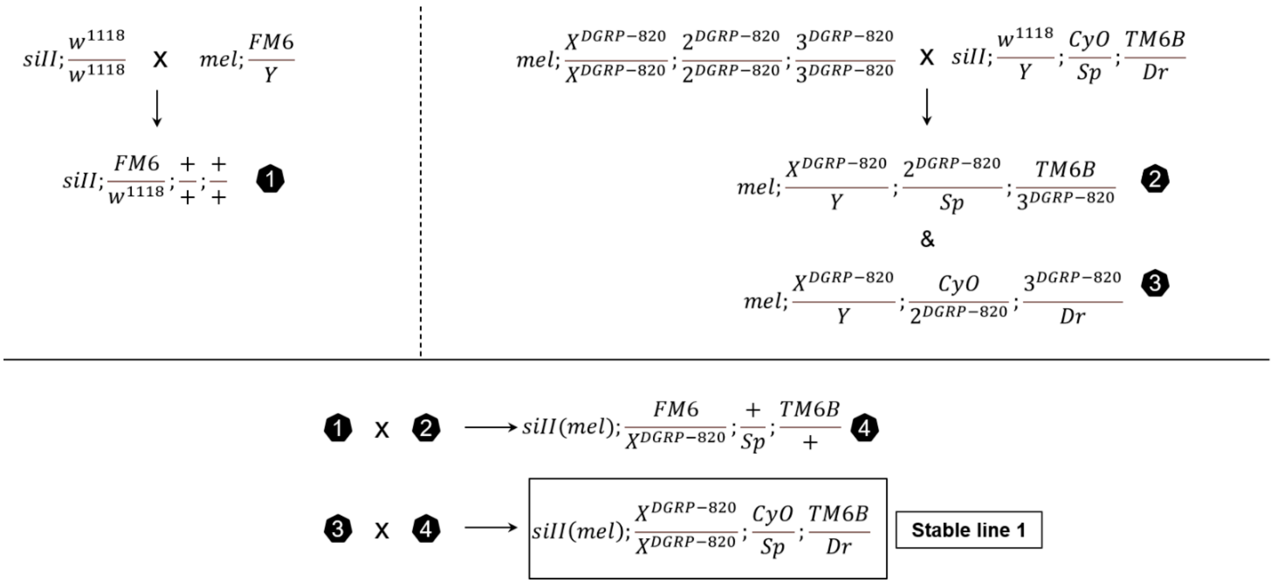

Figure S1: Crossing scheme for the replacement of the *X* chromosome in a w^{1118} background by the *X* chromosome from the *DGRP-820* nuclear background. The crosses were performed twice, once with a *sm21;*w^{1118} line (shown here), and once with a *siI;*w^{1118} line (not shown).
