# Supplementary material for: NUMTs Can Imitate Biparental Transmission of mtDNA—A Case in Drosophila melanogaster
Source: Genes (Basel). 2022 Jun 6;13(6):1023. doi: 10.3390/genes13061023 (PMC9222939; doi:10.3390/genes13061023)
Supplement: Supplementary file 1 [file genes-13-01023-s001.zip › Figure S2.pdf]

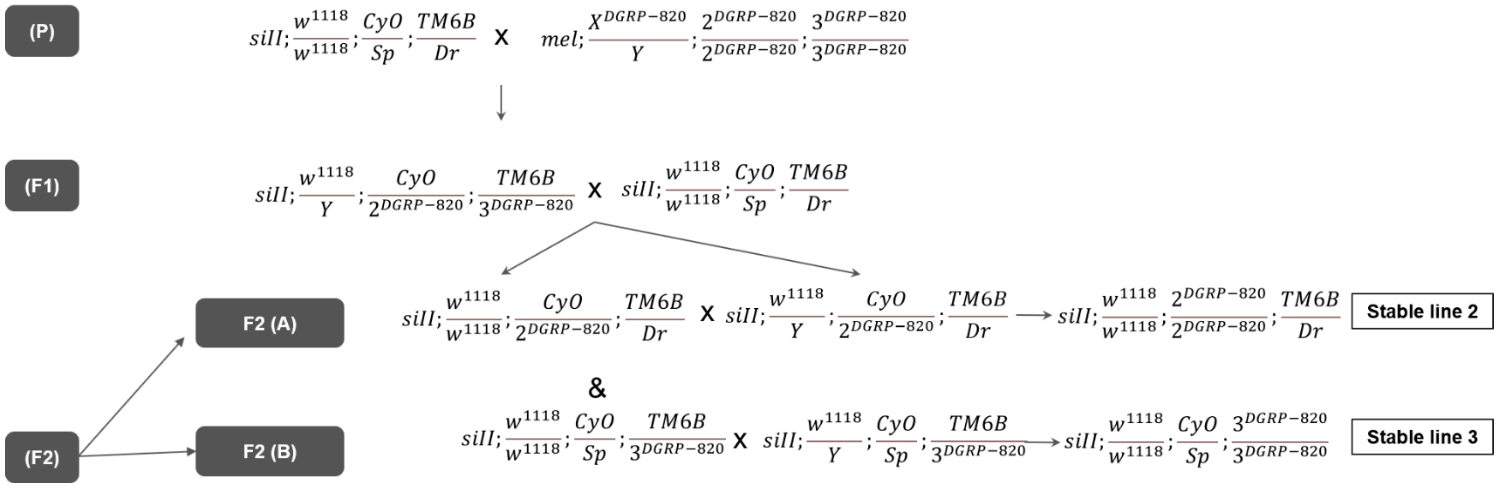

Figure S2: Crossing scheme for the replacement of the autosome chromosomes in a *w[1118]* background by the autosome chromosomes from the *DGRP-820* nuclear background. The crosses were performed twice, once with a *sm21;w[1118]* line (shown here), and once with a *siI;w[1118]* line (not shown).
