# Supplementary material for: NUMTs Can Imitate Biparental Transmission of mtDNA—A Case in Drosophila melanogaster
Source: Genes (Basel). 2022 Jun 6;13(6):1023. doi: 10.3390/genes13061023 (PMC9222939; doi:10.3390/genes13061023)
Supplement: Supplementary file 1 [file genes-13-01023-s001.zip › Figure S3.pdf]

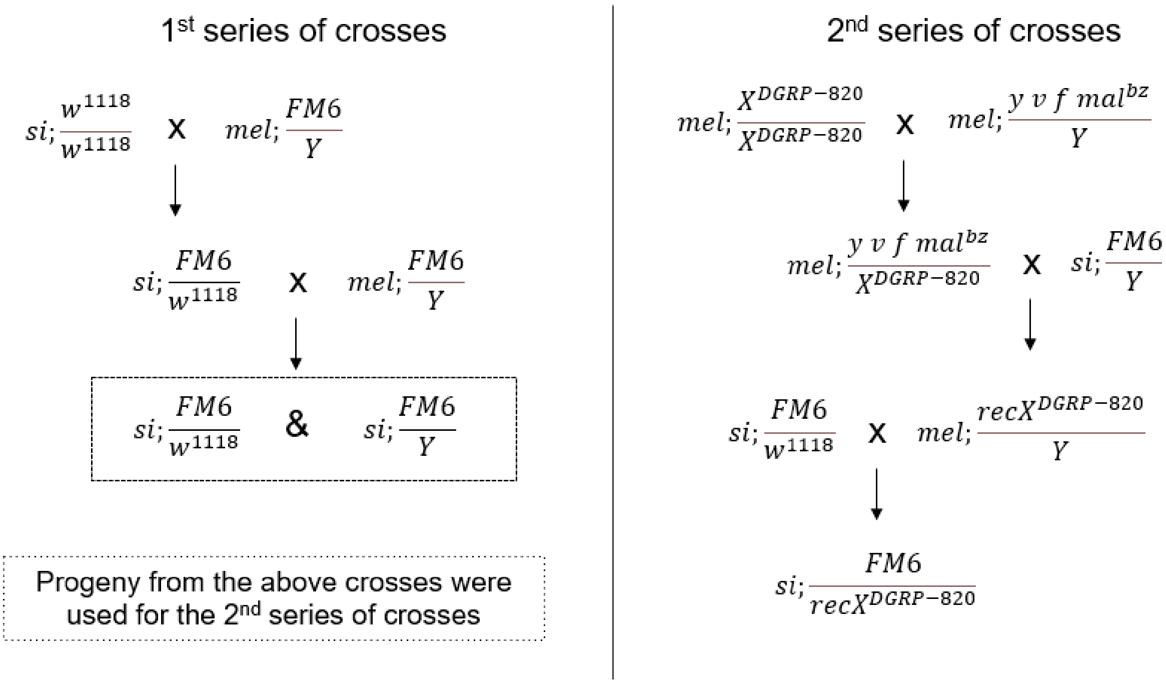

Figure S3: Crossing scheme for mapping the loci responsible for heteroplasmy. The progeny *siIII;FM6/recX*$^{DGRP\text{-}820}$ were phenotyped to determine which *X*$^{DGRP\text{-}820}$ regions they contained. The individuals were then PCR-tested for the presence of the *mel* mitotype.
