# Supplementary material for: NUMTs Can Imitate Biparental Transmission of mtDNA—A Case in Drosophila melanogaster
Source: Genes (Basel). 2022 Jun 6;13(6):1023. doi: 10.3390/genes13061023 (PMC9222939; doi:10.3390/genes13061023)
Supplement: Supplementary file 1 [file genes-13-01023-s001.zip › Figure S4.pdf]

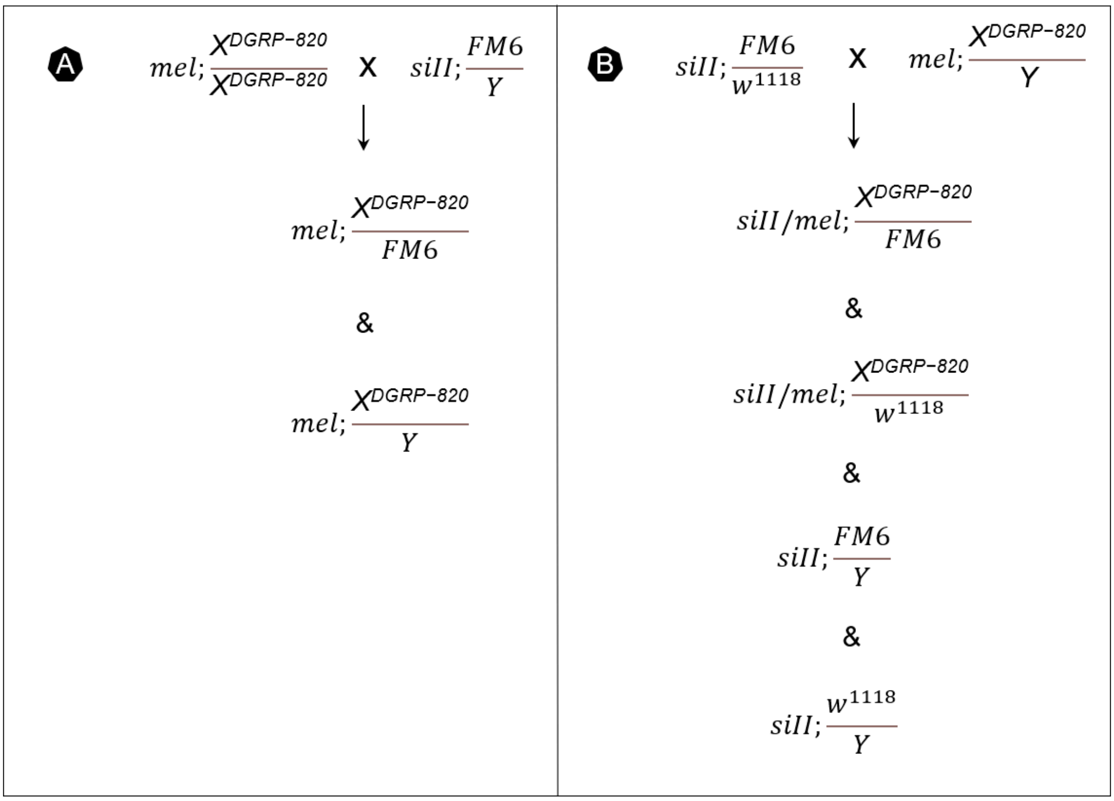

Figure S4: Crosses performed to investigate whether the observed, putative heteroplasmy was created once or whether it was emerging at every generation through paternal leakage. The genotypes of the progeny produced after each cross are shown. **(A)** All progeny were homoplasmic for the maternal mitotype, while no paternal mtDNA leakage was observed. **(B)** Progeny that carried the $X^{DGRP\text{-}820}$ carried also both the *mel* and *siII* mitotypes.
