# Supplementary material for: NUMTs Can Imitate Biparental Transmission of mtDNA—A Case in Drosophila melanogaster
Source: Genes (Basel). 2022 Jun 6;13(6):1023. doi: 10.3390/genes13061023 (PMC9222939; doi:10.3390/genes13061023)
Supplement: Supplementary file 1 [file genes-13-01023-s001.zip › Figure S5.pdf]

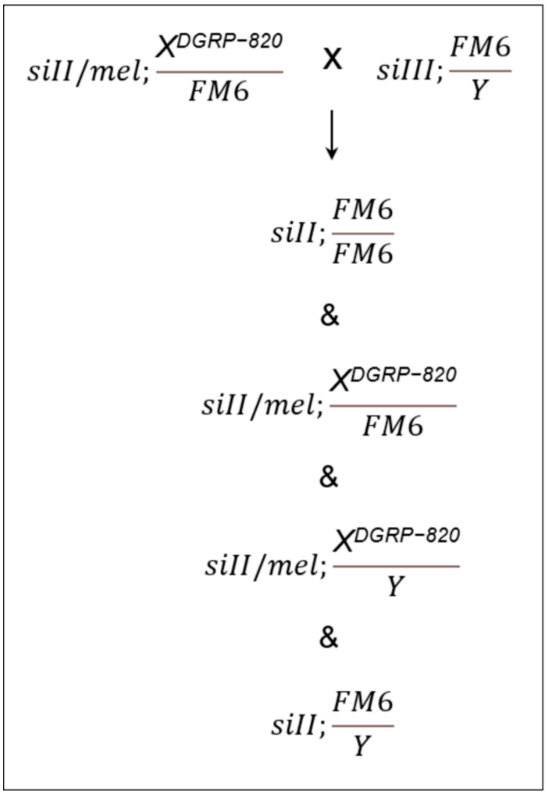

Figure S5: Crosses performed to show whether the putatively heteroplasmic females produced by the crosses from Figure S4-B could transmit their presumed heteroplasmy to the next generation. All progeny that inherited a $X^{DGRP\text{-}820}$ from their mothers had also inherited both maternal mitotype sequences (*siII* and *mel*). On the contrary, the rest of the progeny inherited only the *siII* mitotype.
