# Supplementary material for: NUMTs Can Imitate Biparental Transmission of mtDNA—A Case in Drosophila melanogaster
Source: Genes (Basel). 2022 Jun 6;13(6):1023. doi: 10.3390/genes13061023 (PMC9222939; doi:10.3390/genes13061023)
Supplement: Supplementary file 1 [file genes-13-01023-s001.zip › Figure S6.pdf]

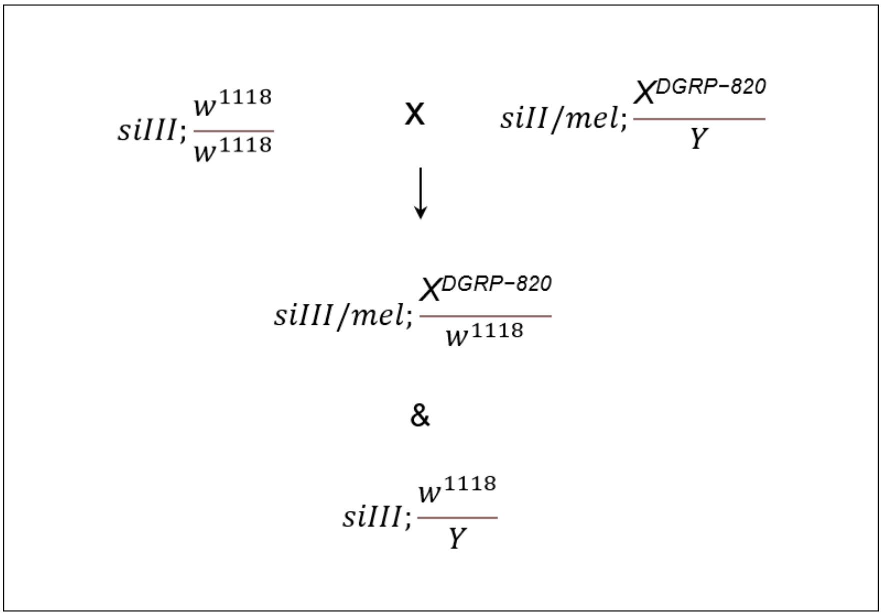

Figure S6: Cross performed to test whether males, putatively heteroplasmic (*mel* and *siII*), with $X^{DGRP\text{-}820}$ chromosome could transmit both their mitotypes to the next generation. Only the *mel* mitotype was transmitted from father, and exclusively to the progeny who also inherited the $X^{DGRP\text{-}820}$. The same cross was repeated with *siI/mel;* $X^{DGRP\text{-}820}/Y$ giving the same results (not shown).
